# The organization of the movement depends mainly on the anticipation of its sensory and emotional consequences

**DOI:** 10.1038/s41598-021-03413-6

**Published:** 2022-02-03

**Authors:** S. Vernazza-Martin, C. Ferrel-Chapus, L. Fautrelle, L. Lachaud, V. Dru

**Affiliations:** 1 Laboratoire des interactions Cognition, Action, Émotion - LICAÉ, UFR STAPS, Université Paris Nanterre, 92000 Nanterre, France; 2 ToNIC, Université de Toulouse, Inserm, UPS, Toulouse, France; 3 EIAP, Département STAPS, Institut National Universitaire Champollion, Campus de Rodez, Rodez, France

**Keywords:** Psychology, Human behaviour, Neuroscience, Emotion, Motor control, Sensorimotor processing

## Abstract

Two sources of emotions influence directed actions, namely, those associated with the environment and those that are consequences of the action. The present study examines the impact of these emotions on movement preparation. It invokes theories from psychology, i.e., ideomotor theory and motor control's cognitive approach through movement analysis. In addition to their action readiness, emotions related to the environment can interfere with actions directed towards a goal. However, intentional action involves a goal that will cause satisfaction when achieved. While most studies consider each emotion's influence separately, few studies confront them to study their respective impact. In the current study, thirty-two right-handed young adults reach for a left target with a stylus that will reduce or enlarge an emotional picture that is initially present (nontarget stimulus). Kinematic analyses show that anticipating the pointing's emotional consequences impacts the final pointing position. All other results emphasize the impact of reducing or enlarging on the preparation and control of movement depending on the direction of movement. The emotional consequences of the action is a weighting factor that is relevant to the action goal and subject's intention, but it is less important than the action's visual consequences.

## Introduction

Individuals commonly use their arm to accomplish various activities and interact with the environment. Due to their daily use, these activities do not seem to imply any difficulty. Nevertheless, they involve complex processes, including visuomotor mechanisms coordinated in a global goal-directed action through an internal model where the consequences of the decision to move could be fully represented^1^. This internal model also allows an early regulation of movement called "impulse control" using the movement's expected sensory consequences. It is used for movement planning and occurs from the beginning of the action during the "initial impulse" phase^2–4^. Thus, these works constitute a significant advance in understanding the intervention of internal models involved not only in movement planning and control but also in the estimation of the sensorial consequences of this movement^5–9^.

However, all this research integrates only sensorimotor aspects of the movement organization (desired trajectories, synchronization of the hand and the arm, sensory consequences of the action). We often forget that in daily life activities, a fundamental component of our practical life, namely, motivation and, more precisely, emotions, influence manual movements towards a spatially defined target intended to interact with the environment. For example, performance in various contexts, such as sports, consumption or industrial tasks, requires adapting movement and manual action with emotional constraints. In another field, through consumer behaviours, we can imagine how people act on their mobile device (smartphone), depending on the search for desired objects. Here, we consider emotions as readiness for action supporting motivation^10,11^, where motivation is responsible for the initiation, maintenance, and cessation of an intended behaviour, similar to the appetitive or aversive valence conferred on the goal of the action and/or elements of the environment on which this behaviour is exerted^12^. Thus, the emotionally marked environmental context, as the emotional valence assigned to the action's goal, can influence reaching and aiming movements.

### An emotionally marked environment influences the organization of the action

Regarding the environmental context, through the motivational direction hypothesis (see Gable and Harmon-Jones^13^, 2010 for review), we automatically and spontaneously assign emotional valences to the different stimuli that surround us, thereby triggering immediate and primitive behavioural predispositions to approach or avoid them^14–16^. For example, when participants respond to attitude object stimuli either by pushing or pulling a lever, they are faster to respond to negatively valenced stimuli when pushing the lever away (avoid) than when pulling it towards them (approach). Likewise, they are faster to respond to positive stimuli by pulling than by pushing the lever^17^. In addition to its action readiness^18^, the emotional context can also be a potential source of distraction for motor actions. Indeed, when subjects are asked to press a button when a target appears, De Houwer and Tibboel^19^ showed that subjects are disturbed and thereby alter their performance when an emotional picture is presented simultaneously as the target. They postulate that emotional stimuli draw attention away from the target, which results in performance deterioration. Similarly, using a task-irrelevant distractor paradigm in which a target and a face with or without emotional expressions are presented simultaneously, Ambron and Foroni^20^ showed that reaching trajectories veer towards faces with emotional expressions but not towards neutral expressions.

### Anticipated emotion associated with the action influences the organization of the action

In addition to this emotional impact on the movement organization, Eder and his colleagues suggested that the link between emotion and movement is built through a representational system of the emotional consequences of action^21–23^. Emotions felt by an individual interacting with an object are linked not only to the object’s valence but also to the positive or negative consequences of his or her action on the object. Therefore, the emotion that the future action will produce promotes behaviours, while affective stimuli that match the affective consequence in valence facilitate response selection^24,25^. For example, associating the push of a button with positive (emergence of a word) or negative (disappearance of a word) consequences produces some affective compatibility effects; the reaction time (RT) is shorter when consequences of a behaviour are congruent with the valence of the presented stimulus (pleasant/unpleasant). In this way, the emergence of a pleasant word or disappearance of an unpleasant word is congruent; the subject performs the movement more quickly, thus reflecting on the facilitation of the organizing processes of the action. Conversely, the emergence of an unpleasant word and disappearance of a pleasant word is incongruent; movement is disturbed, thereby reflecting disruption of these processes^26^. In line with the ideomotor account of action control^27^, the sensorimotor experience allows the creation of maps of perceptual events associated with maps of motor characteristics according to the principle of affordance. These maps constitute a common global system for the perception and planning of action. When an intention to act appears, the characteristics coding the event are weighted according to their relevance to the objective, the subject's intention, and the affectively congruent outcome. Emotions can be considered perceptual representations of interoceptive events^22^. In applied contexts such as navigating across various screens on a mobile device, it is obvious that consumers will act depending on their voluntary and selected search of perceptual objects. In the domain of collective sport performance such, manual strategies are affected by complex spatial configurations about partners (positive cues) and opponents (negative cues) to adopt the best action.

### The organization of action through emotionally marked environment versus anticipated emotions: the contribution of movement analysis

This integration of emotions into the ideomotor approach promotes the role of a multimodal representation of the action in movement planning and/or programming.

Although individuals use highly complex cognitive processes to organize their actions, such as a representation including the resulting sensorial feedback and emotions, we have previously seen that they remain nevertheless dependent on the emotionally marked environment. What is the part of these complex cognitive processes compared to the impact of the emotionally marked environment in the movement organization?

Thus, the general aim of the present study was to identify using motion analysis whether the emotionally marked environment influences the processes that organize and modulate intentional movement or whether this organization is more dependent on the representation of the emotional consequences of action. In this way, movement analysis is a privileged tool to be able to make this distinction, which makes it possible to convoke theories from psychology in the field of neurosciences and, more particularly, in the study of motor control. It makes it possible, in fact, to quantify these two potential emotional effects on various kinematic parameters that reflect the cognitive (cortical) and sensorimotor (subcortical) processes involved in the organization of the action^28,29^. Thus, planning involves cognitive processes that allow us to determine the goal, orient attention to the goal, develop an abstract movement trajectory and make the decision to act. In this context, the kinematic parameters linked to the initial impulse, the trajectory of the movement and goal achievement are indicators of the functioning of these processes. Likewise, movement programming triggered by cognitive processes calls on sensorimotor processes that configure the spatiotemporal components of the motor program. Thus, the duration, amplitude, and mean or maximum movement velocity account for these processes. Finally, an additional study of a late movement regulation, called the “homing phase”, makes it possible to quantify these two potential emotional effects on online motor control, thereby allowing us to adjust the final trajectory to reach the goal^30^.

### The present research

Herein, the task is to perform an intentional arm movement to point with a stylus to a target that appears on the screen to reduce or enlarge an emotional picture that is initially present. From a motor point of view, enlarging a picture simulates approach behaviour, while reducing a picture simulates avoidance behaviour. In agreement with the motivational direction hypothesis, the congruence between the valence of the stimulus and the behaviour makes this behaviour easier. In this way, approaching a pleasant stimulus or avoiding an unpleasant stimulus is positive and facilitates behaviour. We, therefore, consider that anticipating the approach of a pleasant picture or the avoidance of an unpleasant picture is associated with positive effects. Conversely, anticipating the approach of an unpleasant picture or the avoidance of a pleasant picture is associated with negative effects. Moreover, in the same vein as Eder’s works (Eder et al.^21^) and establishing a congruence and incongruence relation between ‘‘turning OFF/ON” and a “negative/positive word”, enlarging a pleasant picture and reducing unpleasant one was considered congruent with a positive consequence while reducing pleasant picture and enlarging unpleasant one was considered incongruent with a negative consequence.

The emotional picture enlargement or reduction that will emerge is known by the participants before the pointing of the target. Participants will be affected by two emotional matters: emotions linked to the stimulus (pictures) and anticipated emotions linked to the consequences of future action. These two emotional matters will either match (pleasant picture/enlargement and unpleasant picture/reduction) or mismatch (pleasant picture/reduction and unpleasant pictures/enlargement) when involving an emotional conflict.

In agreement with the motivational direction hypothesis and with De Houwer and Tibboel^19^ and Ambron and Ferroni^20^, we consider that emotional context can be a potential source of distraction that can interfere with actions directed towards a goal. In this way, the emotional picture could influence the processes that organize and modulate the organization of pointing movement regardless of the emotional action consequences. The second hypothesis is linked to the ideomotor approach (see Shin et al.^31^ for review) We suppose that the affective compatibility between the picture and the anticipated consequences of the arm movement would have the most significant effect on the processes that prepare and control the movement. A positive emotional consequence of the action (the enlargement of pleasant picture or the reduction of unpleasant picture) would facilitate cognitive and sensorimotor processes organizing arm movement. In contrast, a negative emotional consequence (the enlargement of unpleasant picture or the reduction of pleasant picture) would disturb it.

## Results

Thirty-two right-handed young adults were asked to reach for a target with a stylus that appeared at the top left to the screen of a graphic tablet, at the same time as an emotional picture (nontarget stimulus) appeared centred at the top of the screen. Reaching the target provoked the reduction or enlargement of the emotional picture. One group (n = 16) enlarged a pleasant picture or reduced an unpleasant one, which corresponded to positive consequences of the action, while the other group (n = 16) enlarged an unpleasant picture or reduced a pleasant one, which corresponded to negative consequences.

### Valence and arousal ratings

Univariate analyses showed a main effect of the subsets of pleasant and unpleasant pictures on the valence and arousal ratings [*F*(1,62) = 765.6, *p* < 0.001; *ηp*^2^ = 0.93, and *F*(1,62) = 19.2, *p* < 0.001; *ηp*^2^ = 0.24, respectively]. The valence ratings significantly differed between the two categories (unpleasant: 2.31 ± 0.67, pleasant: 6.71 ± 0.6) as arousal ratings were higher for unpleasant pictures (5.9 ± 1.6) than for pleasant pictures (4.2 ± 1.5).

### Temporal parameters

#### Simple reaction time (RT)

**Figure 1:**
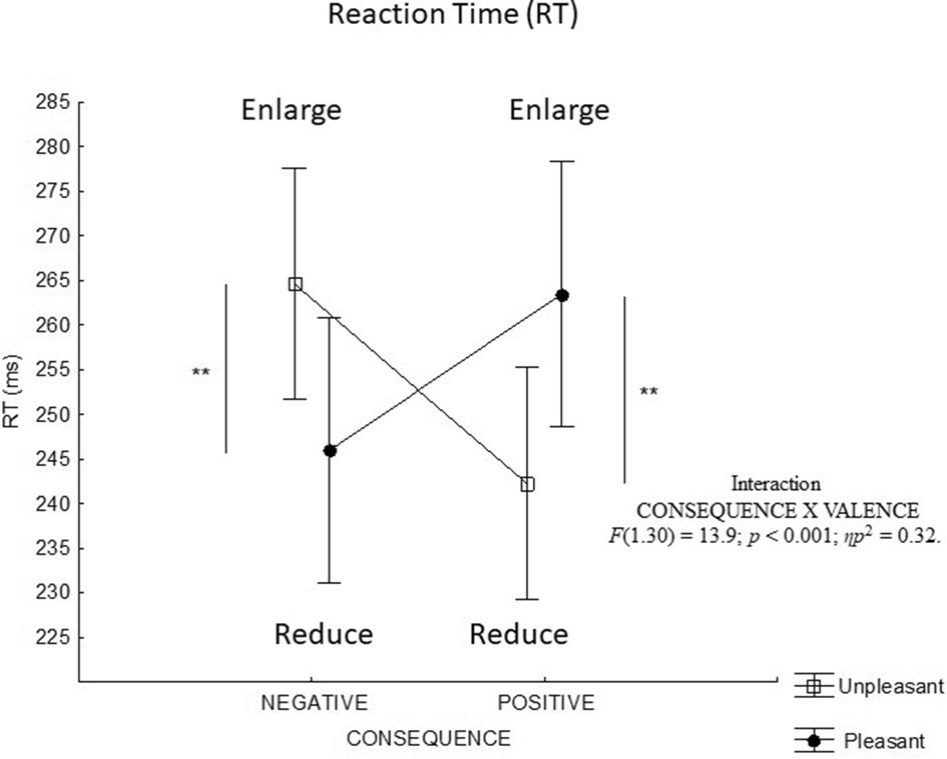
Test sequence and setup for the left-pointing movement: **(a)** Representation of the experimental procedure. The symbol + (or −) indicates enlargement or reduction, respectively. The click on the symbol triggers the occurrence of an emotional picture (nontarget) and the target after 150 ms. The click on the target triggers the enlargement or reduction of the picture for 3 s. **(b)** Typical stylus velocity profile allowing the determination of the temporal parameters of the pointing. **(c)** Typical trajectory of the stylus allowing us to determine the spatial parameters of the pointing.

ANOVA of the RT data showed a significant interaction between action consequence and picture valence [F(1,30) = 17.4; *p* < 0.001; *ηp*^2^ = 0.37]. Therefore, we calculated the exact power of the analysis with an effect size of 0.37. The sensitivity analysis (conducted via G*Power Software, Faul et al., 2007, with Cohen’s recommendations, 1988), with a sample of 32 participants (16 women and 16 men, aged 22–24 years; women average age: 22 ± 1.7; men average age 24 ± 2) with one within-subjects and one between-subjects factor, indicated that an effect size of 0.37 would provide 98% power in regard to detecting a significant effect at a p value of 0.05. The post-test indicated that the RT was shorter for unpleasant pictures than for pleasant pictures when the consequence of the action was positive (p < 0.01), while it was shorter for pleasant pictures than for unpleasant pictures when the consequence of the action was negative (p < 0.01) (Fig. 1).

#### Duration of the initial impulse and the homing phase

There was no effect of the action consequence or picture valence factors [respectively F(1,30) = 0.17; *p* = 0.69 and F(1,30) = 0.33; *p* = 0.57].

#### Movement time (MT)

ANOVA of the MT data showed no effect of the action consequence or picture valence factors [F*f*(1,30) < 1, 0.06; *pf* > *0.8*].

### Spatial parameters

#### Absolute angular error at the end of the initial impulse and the end of the movement (AE)

**Figure 2:**
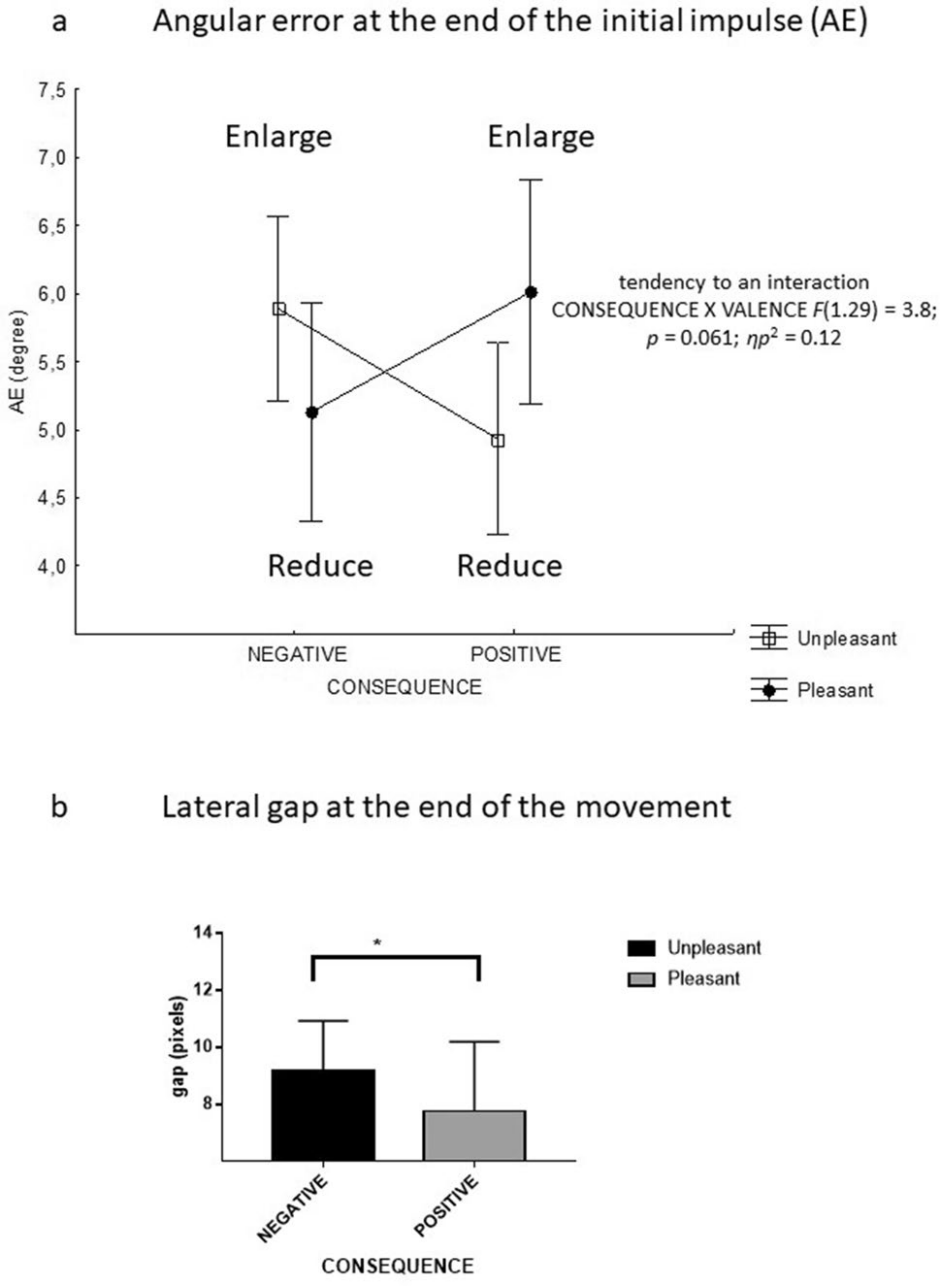
Mean reaction time(s) according to the emotional valence of the pictures and the movement's emotional consequences. A negative consequence for a pleasant or unpleasant picture corresponds to a reduction or an enlargement, respectively. A positive consequence for a pleasant or unpleasant picture corresponds to an enlargement or a reduction, respectively. **p < 0.01.

ANOVA indicated a tendency towards an interaction between action consequence and picture valence only at the end of the initial impulse [F(1*,*29) = 3.8; *p* = 0.061; *ηp*2 = 0.12]. The angular error at this time tended to be greater for unpleasant pictures than for pleasant pictures when the consequence was negative, while it tended to be less important when the consequence was positive (Fig. 2a).

#### Y absolute gap at the end of the initial impulse and the end of movement

ANOVA indicated a main effect of action consequence only on the gap between the stylus relative to the target's centre at the end of the movement [F(1,29) = 5.4; *p* < 0.05; *ηp*^2^ = 0.16]. The means indicated that this gap was more important when the consequence was negative (7.7 ± 2.4 pixels) than when it was positive (9.1 ± 1.8 pixels) (Fig. 2b).

#### Mean variation and maximal deviation of the subject’s trajectory (mVa and max dev, respectively)

**Figure 3:**
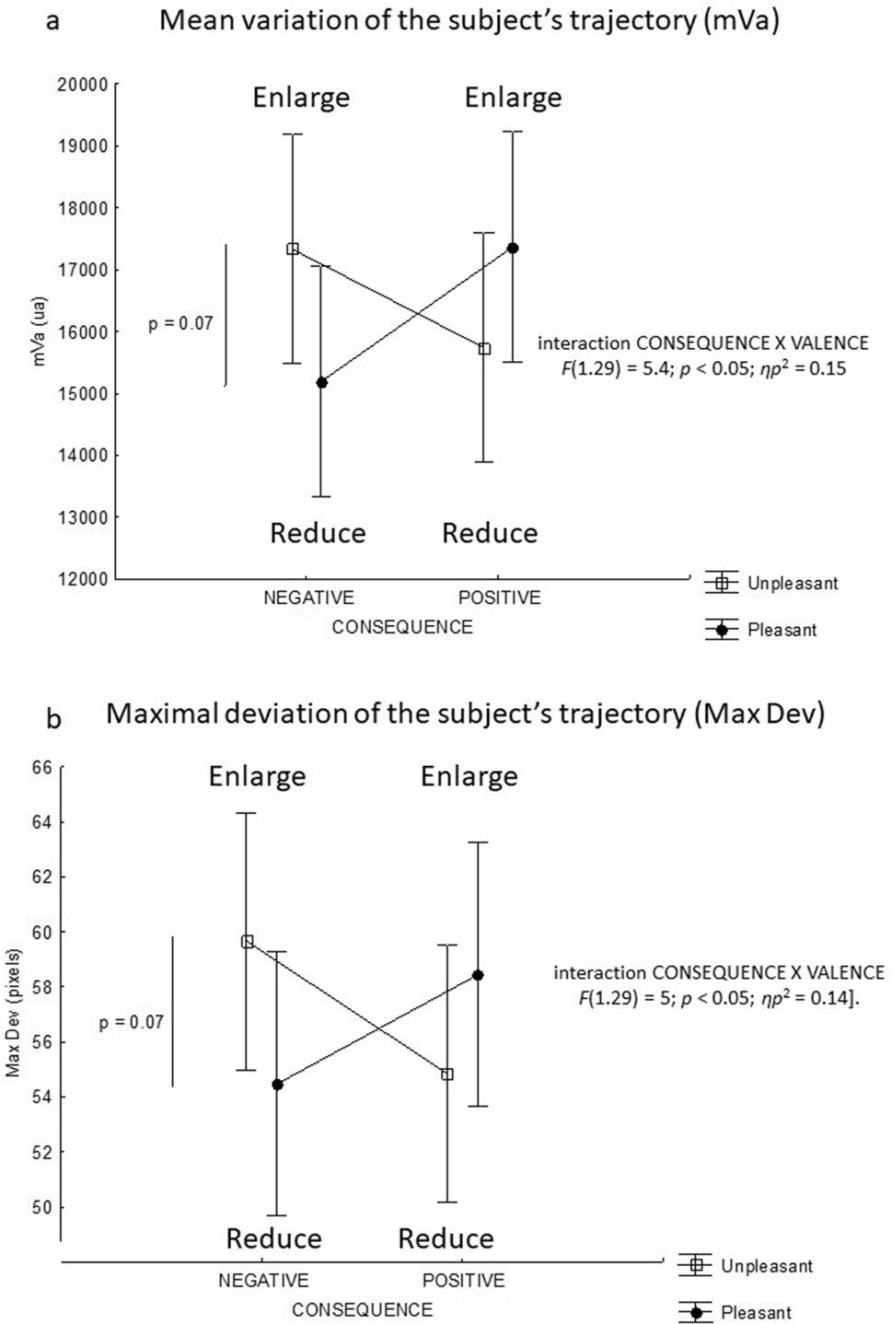
Spatial parameters**.** **(a)** Mean absolute angular error of the trajectory at the end of the initial impulse. A negative consequence for a pleasant or unpleasant picture corresponds to a reduction or an enlargement, respectively. A positive consequence for a pleasant or unpleasant picture corresponds to an enlargement or a reduction, respectively. p = 0.061 indicates a tendency towards an interaction between consequence and valence. **(b)** Mean absolute lateral gap between the stylus and the centre of the target at the end of the movement. *p < 0.05.

There was a significant interaction between action consequence and picture valence for the two parameters [respectively F(1,29) = 5.4; *p* < 0.05; *ηp*^2^ = 0.15 and F(1,29) = 5; *p* < 0.05; *ηp*^2^ = 0.14]. Globally, the mean variation and the maximal deviation of the trajectory tended to be greater for the unpleasant picture relative to the pleasant picture when the consequence was negative, while it tended to be less important when the consequence was positive. Nevertheless, the post-tests only indicated that the variation tended to be shown as greater variation and maximal deviation for the unpleasant picture when the consequence was negative (p = 0.07) (Fig. 3a,b).

## Discussion

The present study aimed to identify, using motion analysis of a pointing task to the left, whether the emotionally marked environment influences the processes that organize and modulate the organization of intentional movement or whether the latter is dependent on the representation of the emotional consequences of action. This approach more finely analyses motor behaviour in its relations with emotions. It mobilizes both ideomotor theory from psychology and motor control theory from behavioural neurosciences. Nevertheless, analyzing the kinematics of a movement involves long and complex processing of several types of data, necessary to characterize the movement precisely. Thus, studies using kinematic analysis generally involve a relatively small number of subjects, which is a limitation. In the present study, the small sample size (16 participants per group) has no consequence on the results concerning the RT (statistical power of 98%). Still, the power of kinematic parameters remains modest, characterized by a significant intra- and interindividual variability. These results should therefore be taken with caution.

Three global results are highlighted in this study. The first is that we observed no valence effect on any of the parameters studied. The valence attributed to a nontarget stimulus does not seem sufficient to influence one when reaching for a target. Movement planning and programming are not organized based on the emotionally marked environment.

The second result is that the emotional consequence of the action alone affects the stylus's final lateral position; this parameter is more distant from the centre of the target when the consequence of the action is negative relative to positive. This result implies that action is associated with its emotional effect and that the anticipation of this effect can increase or decrease efficiency by facilitating or disturbing cognitive processes involved in planning the trajectory endpoint. It also implies that online control does not entirely compensate for this disturbance of the cognitive processes during the homing phase and that the anticipation of the negative emotional consequence of the action also makes online control less effective.

The third result is that an interaction between the emotional valence of the non-target stimulus and the action's emotional consequences appears on practically all the parameters. These interactions seem to bring out a main effect of the action (enlarge/reduce). Indeed, concerning the temporal parameters, the RT analysis indicates that this parameter is shorter for the unpleasant picture than the pleasant one when the consequence is positive (shorter RT when subject will reduce unpleasant picture than he will enlarge pleasant one). We observe the same result for the pleasant picture relative to the unpleasant one when the emotional consequence is negative (shorter RT when subject will reduce pleasant picture relative to enlarge an unpleasant one). Concerning the spatial parameters, the statistical analysis shows that reducing a pleasant or unpleasant picture tends to cause a minor angular error at the end of the initial impulse, i.e., a less variable trajectory with a lower maximal deviation relative to enlarging a pleasant or unpleasant picture. In other words, the cognitive processes involved in action readiness (movement planning and selection of motor program) would be faster and more precise when the subject must reduce the picture than when he or she must enlarge it, regardless of the picture's valence and the emotional consequence of the movement. These latest results point in the same direction and indicate that it would be only reducing or enlarging that affects the processes that organize the action. This could constitute a limiting condition for the emotional impact on motor behaviour. However, this main effect of enlarging/reducing cannot be definitively established using the present experimental protocol. Indeed, we considered an affective compatibility effect between the action and the valence of the stimulus based on the work of Eder et al.^21^. This study is, therefore, interested in a situation of congruence corresponding to the association between enlarging a pleasant picture and reducing an unpleasant one compared to a situation of incongruence (enlarging an unpleasant picture and reducing a pleasant one).This experimental choice had two consequences. The first was that the action of enlarging/reducing was not dissociated from the emotional valence of the pictures, such that the valence factor was partly integrated into the action's emotional consequence factor. The second was that emotional arousal and valence of the action's consequence were not formally assessed. Future research will remove this ambiguity by having two new hypotheses corresponding to two possible interpretations of the present results. The first one would be that the emotions associated with the environment and the emotions associated with the action consequences coexist and interact in a natural situation. This interaction would cancel out each of their potential effects on most kinematic parameters on fast visuomotor tasks and further highlight the link between the action and its visual consequences. According to the integrative ideomotor model of approach and avoidance^21,32^, actions would become associated with weighted codes of their sensory consequences and their emotional effects; all these codes guide action selection. In the present experimentation, the visual consequences of enlargement or reduction have a greater weight than the emotional consequences that the action engenders; this highlights a representation of the action mainly based on its perceptual consequences.

The second hypothesis would be linked to a behavioural point of view. Indeed, enlarging the picture simulates the upper body's forward movement towards the target (approach), while reducing the picture simulates moving away (avoidance). On the other hand, the concept of motor fluency and the body-specificity hypothesis explain that a right-handed subject more easily points to a target located on the side of the dominant hand, which causes a tendance to associate rightward space with positive ideas and leftward space with negative ideas^33,34^.

If we combine the idea that "reduction" is associated with "avoidance" and that a right-handed subject tends to associate "good" with "right" and "bad" with "left," then pointing to the left is congruent with picture reduction. Therefore, the congruency between the emotional spatialization of the movement (left/negative) and its visual effect in behavioural terms (reducing/avoidance) would be a characteristic strongly weighted at the representational level. The impact of the emotional consequences of the action on the processes that organize it should be considered in future research on at least two levels, namely, the pleasure/unpleasure of reaching the goal and the pleasure/unpleasure that provides the motor act itself, independent of the goal to be reached.

In agreement with the ideomotor theory and cognitive approach of motor controls, movement planning and online motor control call for an action representation that would correspond to high-level cognitive processes. This representation would be multimodal. It would be underpinned by an internal model that codes and adapts the event by weighting the perceptual, emotional, and motor characteristics according to not only the future action's goal but also the action itself. When the intention is to reduce or enlarge a picture, the emotional component of the action's visual consequences would be the most dominant factor in trajectory planning. Nevertheless, negative emotional consequences linked to reaching the goal could disrupt the planning of goal achievement and online motor control, thereby resulting in a final position more distant from the centre of the target. To conclude, in applied contexts, many every day and professional life behaviours are frankly associated in a complex way with emotional features of events and with intentional manual actions and their consequences.

## Methods

This research took place entirely in the Superior School of Osteopathy research laboratory in Champs-sur-Marne (77).

### Participants

Thirty-two right-handed young adults were voluntarily recruited from the ESO Paris and participated in this study: 16 men and 16 women, aged 18–29 years (men: 24.3 ± 2.1; women: 22.1 ± 1.7). The entire experimental protocol was approved by the ethics committee of the Cognition–Action–Emotion Interactions Laboratory (LICAÉ), (https://licaenanterre.wixsite.com/licae/ethiquedeontologie). This conformed to the Declaration of Helsinki, and all of the participants provided signed informed consent. They presented normal or corrected vision and did not have balance, neurological, or emotional disorders.

### Materials and procedure

Pointing movement analyses were performed using a laptop (HP ProBook 4530s 15-in. screen using Windows 7) and a Wacom Intuos 4® graphic tablet (dimensions 46.2 × 30.5 cm) with a stylus. E-prime 2.0TM software (Psychology Software Tools, Inc., Sharpsburg, PA) was used for the experiment and allowed the execution of the protocol and the recording of the stylus position in x and y on the graphic tablet at a sampling frequency of 100 Hz. Raw data smoothing and filtering were performed with a 10-Hz low-pass Butterworth filter.

The session lasted approximately 45 min and was divided into four phases.

*The familiarization phase* included four trials. This allowed the subject to test the experimental setup and understand the course of the experiment. The subject had to click on a target at the top right or left of the screen from a starting point in the centre and bottom of the screen.

*The first judgement phase* allowed the subject to assess the task difficulty. Subjects answered on a 9-point scale where 0 corresponded to "extremely difficult" and 9 corresponded to "extremely easy." This phase determined the participant's ability to use the stylus and the graphic tablet and perform the pointing movements on the targets at the top right and left of the screen. The judgement phase appeared several times during the experiment.

*The experimental phase itself* included one hundred twenty-eight trials divided into two blocks of 64 trials. Block 1 corresponded to an assimilation phase that allowed the participant to get used to the requested experimental task (to point at a target on the graphic tablet). Block 2 corresponded to the acquisition phase of the experimental task.

Each trial's goal was to perform a pointing task that aimed to either reduce or enlarge an emotional picture that was initially present. People were divided in two groups. Reaching the target for the first group provoked either the enlargement of a pleasant picture or the reduction of an unpleasant picture; thus, a positive consequence while reaching the target for the second group provoked either an enlargement of an unpleasant picture or a reduction of a pleasant picture; thus, a negative consequence.

A total of 128 digitized photographs (64 pleasant and unpleasant per block) were selected from the International Affective Picture System (IAPS). Based on the scores of the computerized 9-point version of the Self-Assessment Manikin (SAM) scale^16^, experimenters choose pictures according to the gender of the participants and according to the potential effects of arousal on posture and movements^35^.

**Figure 4:**
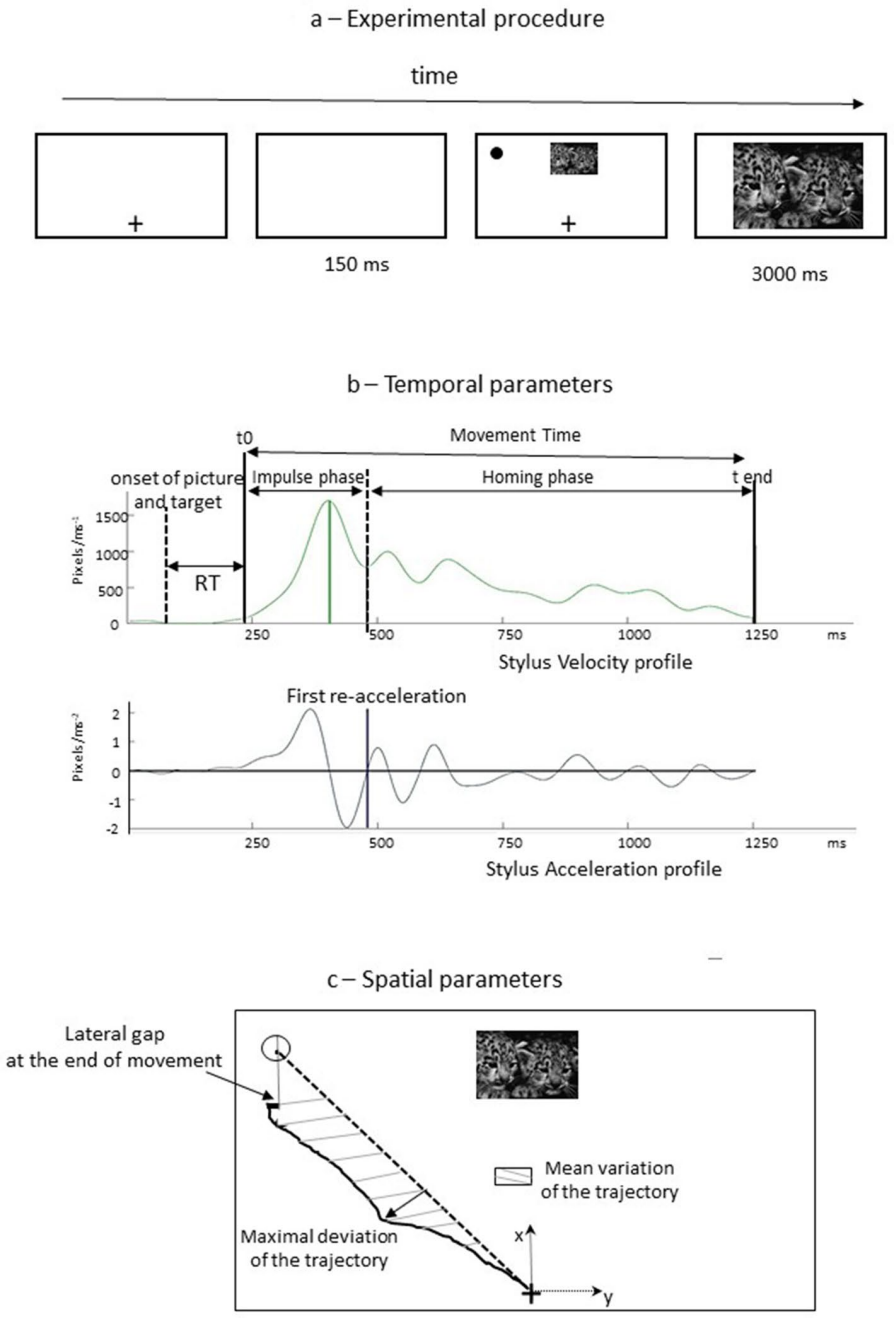
Interaction between action consequence and VALENCE for the mean variation of the trajectory **(a)** and the mean absolute lateral gap between the stylus and the target’s centre at the end of the movement **(b)**. A negative consequence for a pleasant or unpleasant picture corresponds to a reduction or an enlargement, respectively. A positive consequence for a pleasant or unpleasant picture corresponds to an enlargement or a reduction, respectively. p = 0.07 indicates a tendency towards a difference between pleasant and unpleasant pictures when the consequences are negative.

An initial symbol plus " + " or a minus "−" appeared at the bottom and in the middle of the computer screen. This represented the start point of the pointing movement and corresponded to an enlargement or reducing phenomenon of a future picture that would appear centred at the top of the screen. The symbol remained displayed until the participant clicked above. Approximately 150 ms after the "click," a target appeared at the top of the screen on the right or the left side at the same time as the picture (dimension: 256 × 192 pixels). Emotional pictures appeared at random to avoid any learning phenomenon. The subject had to click on the final target to enlarge (1024 × 668 pixels) or reduce (64 × 48 pixels) the picture for 3 s, as indicated by the previous symbol (" + " or "−") (Fig. 4a).

Twenty questions were randomly distributed in the two blocks after clicking on the initial symbol to remind the participants of the action goal and its consequence. The question was "What should you do by clicking on the button?" The subject responded by clicking on the answer box for either "reduce the picture" or "enlarge the picture." The participant's responses were recorded.

*The second judgement phase: *At the end of the experimental phase, the participants had to evaluate the 128 emotional pictures, according to the 9-point scale of the Self-Assessment-Manikin (SAM).

### Coding and preliminary analysis

Experimenters made two analysis choices. First, the analysis was carried out on block 2. This choice was made so that the subject would be accustomed to the material and the experimental protocol. Second, we deliberately chose to analyse challenging movements for a right hander to perform, i.e., left pointing. Indeed, the goal was to emphasize an emotional effect on the kinematics of the movement. Increasing difficulty enhances variability and the sensitivity of the system to potentiate disturbing elements. Here, pointing to the left is more difficult from a biomechanical and behavioural point of view. On the biomechanical side, pointing to the left involves a more articular degree of freedom (3 joints: shoulder/elbow/wrist) than pointing to the right (2 joints: elbow/wrist). On the behavioural side, the concept of motor fluency indicates that a right-handed subject more easily points on the side of his dominant hand^36^.

The processes involved in movement organization—the planning and programming movement processes—were investigated by movement analysis using the following temporal and spatial parameters:

### Temporal parameters (Fig. 4b)

#### Simple reaction time (RT)

Reaction time (RT) was calculated as the time lag between the start of the appearance of the picture and the target on the screen and the movement onset. Movement onset was defined as the moment at which the stylus velocity exceeded 5% of its peak (see Fig. 4b). This time is considered an indicator of the cognitive processes involved in movement planning and triggering motor programs.

#### Duration of the initial impulse

This duration was calculated as the time lag between the start of the movement and the first reacceleration after reaching the maximal velocity (when the acceleration becomes greater than or equal to zero, after reaching the peak velocity). This parameter is considered an indicator of the planning error linked to the subject’s expectations about the availability and salience of different types of feedback^3^; i.e., it provides information about the cognitive processes used to plan movement and triggers motor programs such as early online visual feedback^37,38^.

#### Duration of the homing phase

This phase *corresponds to* the time lag between the first reacceleration after reaching the maximal velocity and the end of the movement, which is defined as the moment at which the stylus velocity exceeds and falls below 5% of its peak (see Fig. 4b). This parameter is an indicator of limb-target control based on sensory feedback.

#### Movement time (MT)

This *is* the time lag between the start and the end of the movement. This parameter accounts for sensorimotor processes involved in movement programming and provides information about online motor control.

### Spatial parameters (Fig. 4c)

#### Absolute angular error at the end of the initial impulse and the end of the movement

This corresponds to the angle in degree between the actual trajectory and the straight path linking the stylus's initial position to the target's centre at the times indicated.

#### Mean variation of the subject's trajectory (mVa)

This parameter was computed as the area under the curve between the straight path linking the stylus's initial position to the centre of the target minus the area under the curve of the stylus trajectory. The results are expressed in units of area (ua) and presented as absolute values.

#### Maximal deviation of the subject's trajectory

The maximum deviation of the trajectory in pixels was quantified using the perpendicular greatest distance between the actual stylus trajectory and the straight path linking the stylus's initial position to the target's centre.

#### Lateral absolute gap at the end of the initial impulse and the end of the movement

This corresponds to the stylus's lateral deviation relative to the centre of the target in pixels, both at the first reacceleration phase after reaching the maximal velocity and at the end of the movement.

These spatial parameters provided indirect information on the cognitive processes allowing movement planning and its trajectory and the sensorimotor processes involved in online movement control.

They were completed by *the mean ratings of the self-reported pictures valance and arousal* corresponding to the mean values obtained for each subject according to the 9-point scale of the Self-Assessment-Manikin (SAM).

### Statistical analysis

The mean ratings of the self-reported pictures’ valence and arousal were compared between each category by univariate analyses.

The experimental design was a 2 (action consequence: negative vs. positive) × 2 (picture valence: unpleasant vs. pleasant), with the variable action consequence manipulated between subjects and the variable picture valence manipulated within subjects. A power analysis (conducted via G*Power Software, with Cohen’s recommendations, 1988)^39,40^, which assumed a medium effect size of 0.25 for the ANOVA with one between-subjects factor and one within-subjects factor, indicated that a total of 34 participants were required to have a 80% power (80% is a minimum required by Cohen, 1988) of detecting a significant effect at a p value of 0.05. The present study included 32 participants. A sensitivity analysis (conducted via G*Power Software and the same recommendations) with a sample of 32 participants (16 women and 16 men, aged 22–24 years; women average age 22 ± 1.7; men average age 24 ± 2) with one within-subjects and one between-subjects factor indicated that an effect size of 0.296 was required to have a 90% power of detecting a significant effect at p value of 0.05, and an effect size of 0.256 was required to have a 80% power of detecting a significant effect at p value of 0.05. The LSD test was used for all other pairwise multiple comparison procedures. Only significant post-tests were analysed. The results are expressed in terms of averages, standard error and the effect size was specified by the partial eta squared (ηp^2^).
